# Advancements in accident-aware traffic management: a comprehensive review of V2X-based route optimization

**DOI:** 10.1038/s41598-025-20878-x

**Published:** 2025-10-08

**Authors:** Hossam M. Zohir, Islam M. Ismael, Eman M. El-Gendy, Mahmoud M. Saafan

**Affiliations:** 1 Mechatronics Engineering Department, Faculty of Engineering, Mansoura University, Mansoura, Egypt; 2 Mechatronics Engineering Department, Faculty of Engineering, New Mansoura University, Mansoura, Egypt; 3 Department of Electrical Engineering, Faculty of Engineering, Mansoura University, Mansoura, Egypt; 4 Faculty of Engineering, Mansoura National University, Mansoura, Egypt; 5 Computers and Control Systems Engineering Department, Faculty of Engineering, Mansoura University, Mansoura, Egypt

**Keywords:** V2X communication, Accident-aware routing, Route optimization, Intelligent transportation systems, Engineering, Mathematics and computing

## Abstract

As urban populations grow and vehicle numbers surge, traffic congestion and road accidents continue to challenge modern transportation systems. Conventional traffic management approaches, relying on static rules and centralized control, struggle to adapt to unpredictable road conditions, leading to longer commute times, fuel wastage, and increased safety risks. Vehicle-to-Everything (V2X) communication has emerged as a transformative solution, creating a real-time, data-driven traffic ecosystem where vehicles, infrastructure, and pedestrians seamlessly interact. By enabling instantaneous information exchange, V2X enhances situational awareness, allowing traffic systems to respond proactively to accidents and congestion. A critical application of V2X technology is accident-aware traffic management, which integrates real-time accident reports, road congestion data, and predictive analytics to dynamically reroute vehicles, reducing traffic bottlenecks and improving emergency response efficiency. Advanced computational algorithms, including heuristic methods, machine learning models, and AI-driven optimization techniques, play a vital role in enhancing routing decisions within V2X networks. By leveraging these algorithms, modern traffic systems can transition from reactive congestion management to proactive traffic optimization, significantly improving urban mobility. Despite its potential, the large-scale deployment of V2X-enabled traffic management systems faces several challenges, including network reliability, data privacy, cybersecurity risks, and interoperability issues. Additionally, concerns related to algorithmic transparency, ethical decision-making, and standardization of V2X communication protocols must be addressed to ensure seamless integration into existing infrastructure. Unlike existing surveys that broadly examine V2X communication or intelligent transportation systems (ITS), this review uniquely focuses on accident-aware traffic management and route optimization. It synthesizes state-of-the-art accident detection methods, routing strategies, and optimization algorithms, while identifying research gaps and proposing future directions for integrating V2X technologies into safer, adaptive, and intelligent transportation systems. By providing these targeted insights, the study contributes to the development of smarter, safer, and more efficient road networks, offering valuable guidance for researchers, policymakers, and industry professionals working to shape the future of urban mobility.

## Introduction

Urban mobility is facing unprecedented challenges due to traffic congestion, rising accident rates, and inefficient traffic management systems. The growing number of vehicles on the road, coupled with unpredictable traffic dynamics, road incidents, and infrastructure limitations, has pushed conventional traffic control strategies to their limits^1^. Traffic congestion not only leads to longer travel times but also results in excessive fuel consumption, increased emissions, and economic losses. Road accidents further exacerbate these issues, causing significant disruptions, delays in emergency response, and safety concerns^2^. Consequently, intelligent, data-driven solutions are essential to improving transportation efficiency, reducing congestion, and enhancing road safety^3,4^.

One of the most transformative innovations in modern transportation systems is V2X communication, which enables seamless real-time data exchange between vehicles and their surrounding environment^5^. Unlike traditional traffic management systems that rely on centralized control and static rules, V2X fosters a dynamic, decentralized, and intelligent transportation ecosystem where vehicles communicate with each other, roadside infrastructure, pedestrians, and cloud-based network services^6^. This interconnected network enhances situational awareness, allowing vehicles and traffic management systems to detect, predict, and respond to traffic incidents in real time, thereby reducing congestion and improving safety^7^.

A key application of V2X technology is accident-aware traffic management, which leverages real-time accident reports, road congestion data, and predictive analytics to dynamically reroute vehicles, minimizing delays and ensuring rapid emergency response^8^. Traditional traffic control mechanisms, which often rely on historical data and pre-defined traffic rules, struggle to adapt to rapidly evolving traffic conditions, such as accidents, road closures, or unexpected congestion. To overcome these limitations, advanced computational techniques have been integrated into V2X networks to optimize traffic flow and improve route planning^9,10^.

Various algorithmic approaches play a crucial role in traffic optimization and decision-making within V2X-enabled ITS. These include optimization algorithms, reinforcement learning models, heuristic-based approaches, and predictive analytics methods^11,12^. While search algorithms such as Dijkstra’s shortest path, A* search, and evolutionary computing methods remain essential for identifying optimal routes, machine learning-based models, deep reinforcement learning, and hybrid AI-driven approaches have gained traction in recent years^13^. These adaptive algorithms consider multiple real-time parameters, such as accident severity, congestion levels, road conditions, vehicle density, and environmental factors, to generate dynamic and intelligent traffic control strategies^14^. By integrating V2X communication with these advanced computational techniques, transportation systems can transition from reactive congestion management to proactive traffic optimization, significantly enhancing urban mobility^15^.

Despite these advancements, several challenges must be addressed to ensure the widespread adoption of V2X-based traffic management systems. Network reliability, data security, privacy concerns, computational complexity, and interoperability remain key obstacles^16–19^. Additionally, the standardization of V2X communication protocols and the integration of heterogeneous data sources are critical for seamless interoperability across vehicles, traffic control centers, and infrastructure providers^20^. As AI-driven decision making becomes more prevalent, concerns related to algorithmic transparency, ethical considerations, and bias mitigation must also be carefully examined^21^.

This paper provides a comprehensive and focused review of accident-aware traffic management within V2X networks, emphasizing the latest advancements, critical challenges, and open research opportunities. Unlike previous surveys that broadly examine V2X communication or ITS, this work specifically links accident detection, real-time routing, and optimization within a unified framework. The main contributions of this study are:

- Focused synthesis of V2X communication technologies, ITS integration, accident detection, routing, and optimization, highlighting their interplay in accident-aware traffic management.
- Critical comparison of prior surveys, showing how existing studies remain fragmented and how this article provides a unified perspective.
- Evaluation of techniques for accident detection, prediction, and routing optimization—including AI-based approaches, heuristic algorithms, and multi-objective models—emphasizing their strengths, limitations, and real-world challenges.
- Identification of open challenges and research opportunities, including scalability, cybersecurity, explainability, data standardization, and ethical considerations, offering a structured roadmap for future studies.

The remainder of this paper is structured as follows: Section “Literature Retrieval methodology” outlines the Literature Retrieval Methodology, detailing the databases consulted, the time span covered, the search strings adopted, and the inclusion and exclusion criteria, supported by a flow diagram. Section “V2X communication technologies” explores the underlying technologies of V2X communication and their significance in modern transportation. Section “Intelligent transportation systems” delves into the role of ITS in enhancing traffic efficiency and safety. Section “Traffic management in V2X networks” examines various traffic management approaches in V2X networks and their implications for real-time control. Section “Accident-aware routing strategies” reviews accident-aware routing strategies and their contribution to proactive incident handling. Section “Algorithms for route optimization” evaluates different algorithmic approaches for optimizing routes and traffic flow. Section “Discussion” provides a discussion of the reviewed literature, highlighting key insights, unresolved challenges, and opportunities for integrating accident-aware V2X systems into future intelligent transportation frameworks. Finally, Section “Conclusion and future directions” presents the conclusions and outlines potential future research directions.

## Literature retrieval methodology

To ensure the comprehensiveness and replicability of this review, a structured literature retrieval methodology was adopted. The process involved selecting relevant databases, defining precise search keywords, and applying inclusion and exclusion criteria to filter studies. A summary of the retrieval process is presented in Table 1, which outlines the databases searched, the keywords employed, and the criteria used for article selection.

**Table 1:** Literature retrieval methodology.

**Aspect**	**Details**
**Databases Searched**	IEEE Xplore, ScienceDirect, SpringerLink, ACM Digital Library, Google Scholar
**Keywords Used**	“V2X communication”, “vehicle-to-everything”, “intelligent transportation systems”, “accident-aware routing”, “traffic management”, “route optimization”
**Publication Period**	2019–2025
**Inclusion Criteria**	Peer-reviewed journal and conference papers Focused on V2X technologies, ITS applications, accident-aware traffic management, or optimization algorithms
**Exclusion Criteria**	Non-English publications, non-peer-reviewed works, Studies focused only on vehicular hardware without communication or routing aspects

## V2X communication technologies

V2X communication encompasses four core interactions—Vehicle-to-Vehicle (V2V), Vehicle-to-Infrastructure (V2I), Vehicle-to-Pedestrian (V2P), and Vehicle-to-Network (V2N)—each designed to enhance road safety, traffic efficiency, and overall transportation systems^22^. V2V enables the exchange of speed, direction, and hazard information to reduce collision risks, while V2I allows vehicles to interact with infrastructure such as traffic lights to improve flow and provide real-time updates. V2P enhances pedestrian safety by alerting drivers to nearby pedestrians, and V2N connects vehicles to broader networks, offering access to cloud services and real-time data essential for autonomous driving^23–26^.

Two primary wireless access technologies dominate current V2X research and deployment. Dedicated Short-Range Communication (DSRC), developed under IEEE 802.11p/WAVE standards, was an early enabler of V2X by supporting Basic Safety Messages (BSMs) to enhance situational awareness^27^. Despite its low-latency benefits, DSRC faces significant challenges, including channel congestion, lack of acknowledgment mechanisms, and susceptibility to interference, which limit scalability in dense environments^28^. In contrast, Cellular-V2X (C-V2X) leverages cellular infrastructure to expand communication range and reliability. Applications include emergency message prioritization^29^ and eco-driving support via traffic signal communication^30^. However, the debate over the most suitable standard—DSRC, C-V2X, or 5G/6G—remains unresolved, creating fragmentation in both research and deployment^31^.

Emerging 5G-based V2X architectures promise ultra-low latency and support for high-bandwidth applications, including Intelligent Perception Systems (IPS) for blind intersections, though trade-offs exist between deployment cost, mmWave capacity, and Sub-6GHz scalability^32^. Alongside these advances, researchers highlight persistent cross-cutting challenges. Security remains a major concern, as increased connectivity exposes V2X systems to cyberattacks, requiring robust cryptographic, intrusion detection, and AI-driven defenses^17,33^. Communication imperfections—such as packet loss, message delays, and inconsistent ordering—can significantly degrade the performance of autonomous intersection control algorithms, necessitating redundancy and standardized testing frameworks^34^. Moreover, edge-assisted motion planning must adapt to latency variations and imperfect channel state information, balancing aggressive and conservative driving strategies to maintain safety^35^.

Taken together, these studies underscore both the promise and complexity of V2X technologies. While DSRC and C-V2X offer foundational capabilities, large-scale deployment continues to face hurdles related to standardization, interoperability, scalability, and resilience to cyber-physical threats. The integration of 5G, edge computing, and blockchain may alleviate some of these limitations, but they also introduce new challenges around cost, energy efficiency, and security. Future research must focus on harmonizing communication standards, optimizing wireless resource allocation, and embedding adaptive AI models to ensure safe, scalable, and resilient V2X-enabled transportation systems.

**Fig. 1:**
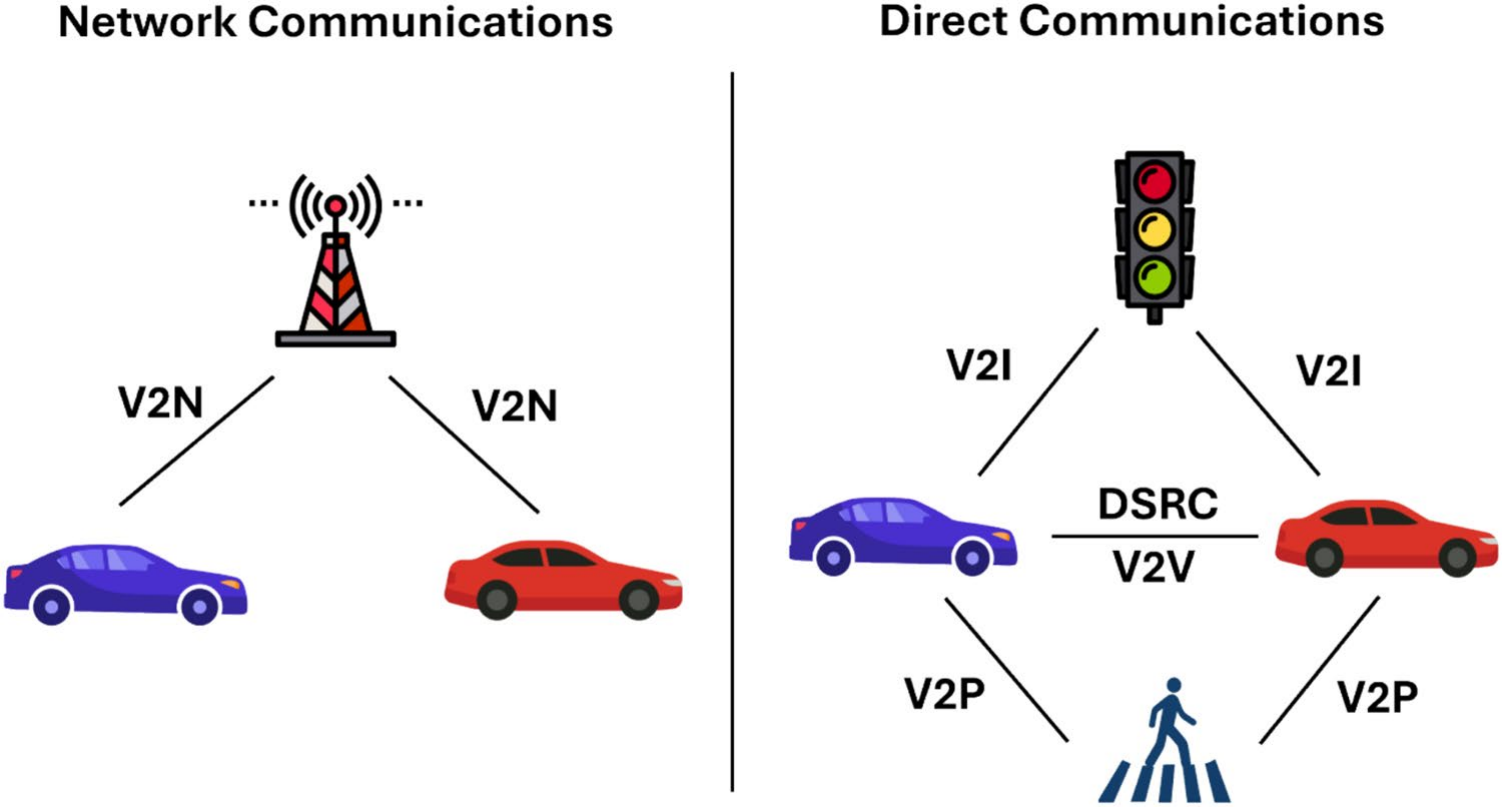
V2X Communication overview.

A comparison of these technologies, their key features, and associated challenges is summarized in Table 2. A comprehensive understanding of V2X communication requires distinguishing between network-based and direct communication methods. Fig. 1 illustrates the distinction between network-based communications and direct communications, emphasizing their respective roles in improving road safety and traffic efficiency.

**Table 2:** V2X communication technologies.

**Reference**	**Study**	**Year**	**Technology**	**Key Features**	**Challenges**
^31^	Clancy et al.	2024	DSRC, C-V2X, 5G	Extends perceptual range of autonomous vehicles, enhances ITS	Lack of unified standard, interoperability issues, network congestion, security vulnerabilities
^27^	Yin et al.	2014	DSRC (IEEE 802.11p)	Supports BSMs, improve situational awareness and road safety	Channel congestion, no handshake mechanism, self-interference issues
^28^	Wu et al.	2013	DSRC (WAVE)	WLAN-based, low-latency vehicle communication	Communication failures in high traffic, no internet access support, message delivery issues
^29^	Nair et al.	2024	C-V2X	Optimized emergency resource allocation	Implementation complexity
^30^	Liang et al.	2024	C-V2X	Enhances eco-driving, reduces traffic stops	Data transmission challenges
^25,26^	Bhargavi et al., Arikumar et al.	2022, 2023	C-V2X	V2V, V2I, and V2P for safety and efficiency	Connectivity reliability
^32^	Clancy et al.	2024	C-V2X (5G NR)	Private 5G network with Sub-6GHz and mmWave for IPS	Deployment cost, bandwidth limitations, data compression trade-offs

V2X technologies play a pivotal role in modern Intelligent Transportation Systems by enabling real-time communication among vehicles, infrastructure, pedestrians, and networks. Their applications extend across multiple domains: safety, where V2V and V2P help prevent collisions and protect vulnerable road users; traffic efficiency, where V2I supports eco-driving, adaptive traffic signaling, and congestion reduction; autonomous driving, where V2N provides access to cloud and edge services for cooperative maneuvers and motion planning; and sustainability, where reduced fuel consumption and optimized traffic flow contribute to greener urban mobility. Emerging technologies such as DSRC, C-V2X, and 5G/6G further enhance these applications by offering low-latency, high-capacity communication for advanced scenarios like intelligent intersection management, coordinated lane merging, and emergency response optimization. The main applications, advantages, and challenges of these V2X technologies are summarized in Table 3.

**Table 3:** Applications of V2X technologies.

**V2X Type/Technology**	**Key Applications**	**Advantages**	**Challenges/Limitations**	**References**
**V2V**	Exchange of speed, direction, and hazard data to prevent collisions	Improves situational awareness; reduces crash risks	Communication delays, packet loss, message order issues can degrade safety	^22,34^
**V2I**	Communication with traffic lights, road sensors, and infrastructure for traffic flow optimization	Real-time traffic updates; smoother mobility (eco-driving); reduced stops	Infrastructure cost, interoperability, dependence on coverage	^23,30,32^
**V2P**	Alerts drivers to pedestrian presence via connected devices	Enhances pedestrian safety	Device dependency; privacy concerns	^23,25^
**V2N**	Connection to cloud services, edge servers, IoT platforms	Enables real-time info sharing; supports autonomous driving; access to advanced analytics	Latency, congestion, scalability of network	^24,33,35^
**DSRC (IEEE 802.11p/WAVE)**	Broadcast of Basic Safety Messages (BSMs); short-to-medium range comms	Low latency; no server required; effective in areas without cellular coverage	Channel congestion; no ACK mechanism; limited scalability	^27,28^
**C-V2X, 4G/5G**	Safety messages, eco-driving, emergency service support, traffic light integration	Higher capacity; supports V2V, V2I, V2P; scalable with 5G	Spectrum allocation, network load, rural coverage	^25,26,29,30^
**5G/6G-based V2X**	High-bandwidth, low-latency communication; intelligent perception at junctions	Supports high-resolution sensing, mmWave capacity, scalable autonomous driving	Deployment cost, bandwidth trade-offs, new attack surfaces	^17,31,32^
**Security in V2X**	Protecting data, authentication, intrusion detection, blockchain integration	Enhances trust, protects privacy, supports resilient networks	High computation cost (blockchain), adaptive key management, adversarial AI risks	^17,33^
**Edge-assisted V2X**	Motion planning, adaptive driving strategies, joint optimization with power control	Reduces collision risks, adapts to real-time delays	Scalability, unpredictable latency in real-world	^35^

## Intelligent transportation systems

In the ever-evolving landscape of urban transportation, integrated and adaptive traffic management systems have emerged as essential solutions to combat congestion, enhance safety, and improve overall efficiency. Various researchers have contributed to this field, each proposing innovative approaches to address modern traffic challenges^36^.

Lupi et al. introduced the LIST Port ITS System, a comprehensive solution integrating traffic video cameras, variable message signs (VMS), and a mobile application. This system provides real-time traffic and noise data, allowing users to select optimal routes to port terminals, ultimately reducing delays and improving travel efficiency^37^.

Building on this concept, Cheng et al. analyzed the impact of the 511 systems implemented across the U.S. Their findings demonstrated a significant reduction in congestion, with an estimated annual saving of over $4.7 billion and 175 million hours. By offering real-time travel information, the system has empowered commuters to make informed decisions, alleviating traffic bottleneck^38^.

Further enhancing safety within traffic management, Smith et al. developed an adaptive system that integrates formal traffic safety rules based on Traffic Conflict Techniques (TCTs). By dynamically adjusting vehicle speeds, this system aims to prevent collisions. The researchers evaluated its effectiveness using traffic flow data from the SR528 highway in Orlando, Florida. Utilizing safety indicators such as time-to-collision and space headway, alongside the Mathematica computer algebra system and the Simulation of Urban Mobility (SUMO) micro-simulation tool, they demonstrated a notable increase in safety margins and a reduction in collision risks^39^.

Zhao et al. took traffic optimization further by incorporating user preferences into an integrated management system designed for large cities. Using connected vehicles (CVs) to estimate traffic conditions, the system generates multi-layer control instructions, optimizing mobility, energy consumption, and driving comfort. Microscopic simulations revealed impressive results, including a 32% reduction in vehicle delay, a 4% decrease in fuel consumption, and a 24% restriction on unnecessary left and right turns. At an optimal market penetration rate, travel time delays dropped by 38%, fuel consumption by 4.5%, and trip distances by 2%. However, limitations were identified, including the assumption of single-user preferences per vehicle and the need for dynamic regional boundaries^40^.

Expanding the scope of intelligent traffic management, Surekha et al. proposed an Intelligent Traffic Management System (ITMS) leveraging advanced technologies such as computer vision, machine learning, and artificial intelligence. By utilizing the YOLO v7 algorithm, the system enforces helmet compliance, detects traffic signal violations, and identifies vehicle number plates through optical character recognition. This automated approach enhances road safety, mitigates accidents, and improves overall traffic regulation^41^.

**Fig. 2:**
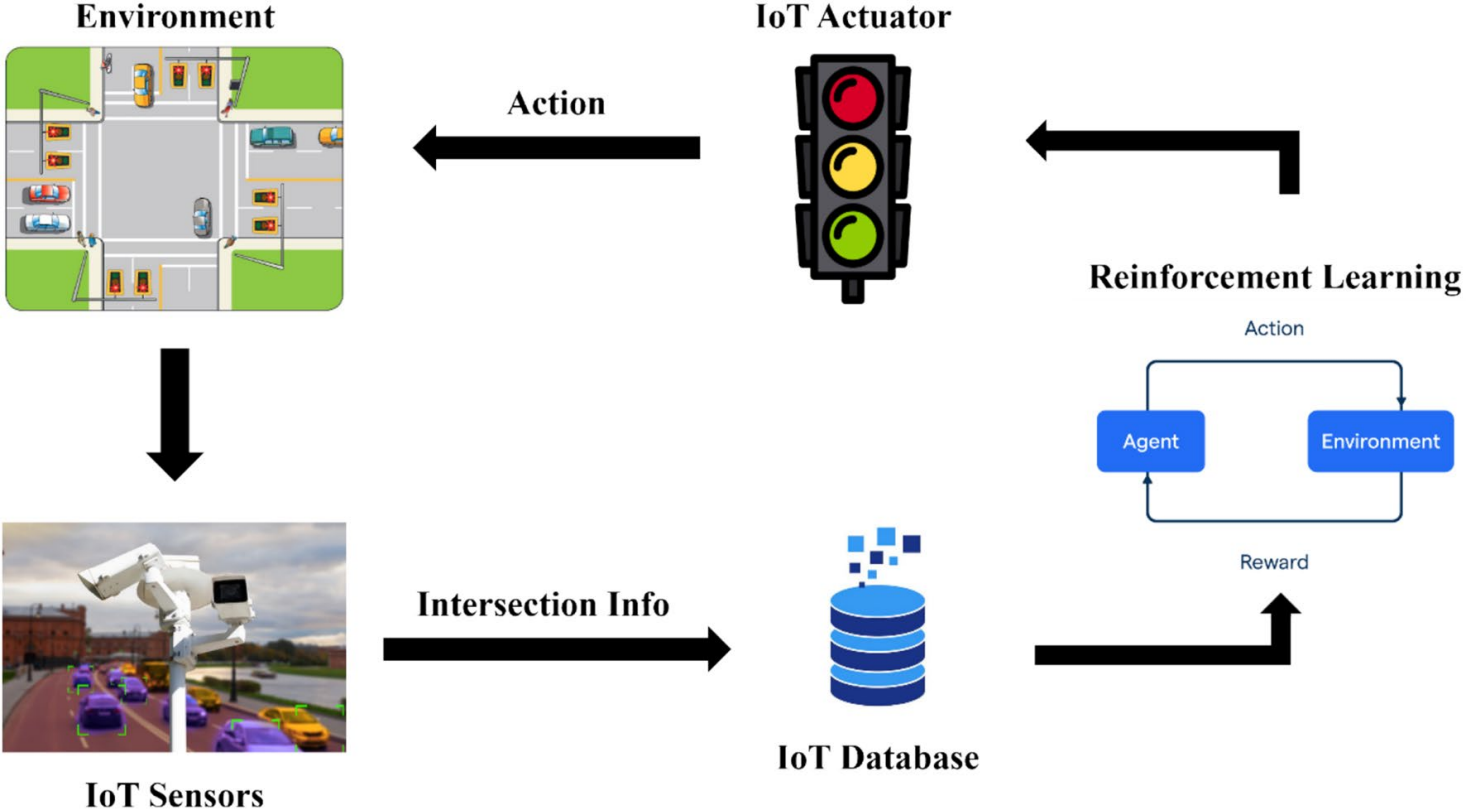
IoT and MARL approach for ATSC.

Recognizing the importance of adaptive control in urban traffic systems, Damadam et al. presented a Multi-Agent Reinforcement Learning (MARL)-based Adaptive Traffic Signal Control (ATSC) system integrated with Internet of Things (IoT) devices to optimize traffic flow in Shiraz City. The system utilizes real-time traffic data from surveillance cameras and sensors to dynamically adjust traffic signals, reducing congestion and improving overall traffic efficiency. MARL enables cooperation between multiple intersections, enhancing decision-making by incorporating local and adjacent intersection data. Simulations conducted on both synthetic intersections and a real-world map of Shiraz City demonstrate that the proposed system significantly outperforms the traditional fixed time scheduling approach, reducing vehicle queue lengths and waiting times. The findings highlight the system’s effectiveness, especially during peak hours, and suggest future expansion to additional intersections while considering pedestrian impact for enhanced traffic management. A schematic representation of the IoT and MARL approach is illustrated in Fig. 2^42^.

Additionally, Nguyen et al. introduced a bi-level control framework for vehicle route optimization, integrating Connected and Automated Vehicles (CAVs) and ITS to enhance traffic flow and reduce congestion. The framework combines system-level traffic flow control with individual vehicle speed control, ensuring optimal fuel efficiency, reduced stops, and improved road safety. A group-based method is proposed to synchronize macroscopic and microscopic traffic models, optimizing vehicular trajectories while maintaining network-wide efficiency. The approach employs Mixed-Integer Linear Programming (MILP) models at both control levels, iteratively solving them to achieve optimal traffic management. Numerical results demonstrate the framework’s effectiveness in minimizing vehicular emissions, reducing queue formations, and improving overall traffic flow^43^.

Incorporating more advanced computational techniques, Jia et al. developed an adaptive traffic signal control method based on Graph Neural Networks (GNN) and the Dynamic Entropy-Constrained Soft Actor–Critic (DESAC) algorithm. This model extracts global and local traffic features, optimizing signal control dynamically. Simulations on the CityFlow platform demonstrated that G-DESAC outperforms traditional methods like DQN, SAC, Max-Pressure, and DDPG, achieving lower delays, shorter queues, and improved throughput. While computationally demanding, this approach offers a robust and scalable solution for traffic control^44^.

Similarly, Wang et al. introduced an adaptive traffic signal control system utilizing offline reinforcement learning (Offline RL) through the SD3-Light model. By dynamically adjusting signal phases and durations based on real-time intersection states, the system reduces reliance on live data, cutting operational costs while improving traffic efficiency. Evaluations on real-world datasets demonstrated significant reductions in average travel time, along with high performance on novel metrics such as destination–arrival average travel time (DATT) and destination–arrival rate (DAR)^45^.

Exploring AI-powered traffic optimization, Patil et al. presented a dynamic signal timing adjustment system based on real-time vehicle density analysis. Their Python-based simulation model indicated that adaptive traffic control leads to reduced travel times, lower emissions, and improved pedestrian safety. The system also contributes to alleviating driver fatigue and ensuring fair access to transportation infrastructure^46^.

Finally, Agrahari conducted an extensive study on Adaptive Traffic Signal Control (ATSC) systems, categorizing various approaches, including Fuzzy Logic (FL), Metaheuristic (MH), Dynamic Programming (DP), Reinforcement Learning (RL), Deep Reinforcement Learning (DRL), and hybrid models. The study highlighted the efficiency of AI-driven ATSC systems in adjusting traffic signals dynamically. However, it also identified a crucial research gap in optimizing multi-intersection ATSC, where coordinated signal control is essential. Future research should explore multi-agent systems capable of handling real-time fluctuations while considering additional real-world factors such as pedestrian movement, weather, and emergency scenarios^47^.

To address these challenges, ITS have been developed, utilizing cutting-edge technologies to improve traffic management and enhance road safety. ITS encompasses a wide range of applications that collect and analyze real-time data from vehicles, infrastructure, and communication networks, with the goal of optimizing transportation efficiency and mitigating the impacts of accidents and congestion^48^.

To provide a clearer comparison of the various intelligent traffic management systems discussed, Table 4 summarizes their key technologies, methodologies, outcomes, and limitations. These systems leverage advanced techniques such as AI, IoT, and reinforcement learning to enhance traffic efficiency, safety, and real-time decision-making.

**Table 4:** Overview of intelligent traffic management systems – innovations, performance metrics, and challenges.

**Study**	**Proposed System**	**Technology Used**	**Key Outcomes**	**Limitations**
Lupi et al.^37^	LIST Port ITS System	Traffic cameras, VMS, mobile app	Reduced delays, improved efficiency	Focused on port terminals only
Cheng et al.^38^	511 Traffic Information System	Real-time travel updates	$4.7B annual savings, reduced congestion	Implementation varies by region
Smith et al.^39^	Adaptive Safety System	TCTs, SUMO, Mathematica	Increased safety margins, reduced collisions	Requires high-quality traffic data
Zhao et al.^40^	User-Preference-Based Optimization	CVs, Multi-layer control	38% delay reduction, lower fuel consumption	Assumes single-user preferences
Surekha et al.^41^	Intelligent Traffic Management System (ITMS)	Computer vision, ML, AI (YOLO v7)	Automated enforcement, improved safety	Limited to specific traffic violations
Damadam et al.^42^	Adaptive Traffic Signal Control (ATSC)	IoT, AI	Reduced queues and wait times	Effectiveness depends on IoT infrastructure
Jia et al.^44^	G-DESAC Adaptive Signal Control	GNN, DESAC Algorithm	Lower delays, shorter queues	Computationally demanding
Wang et al.^45^	Offline RL-Based Traffic Control	SD3-Light Model, RL	Reduced travel time, lower operational costs	Requires high-quality historical data
Patil et al.^46^	AI-Powered Signal Timing	Real-time vehicle density analysis	Improved efficiency, lower emissions	Focused on urban intersections
Agrahari^47^	Adaptive Traffic Signal Control Review	FL, MH, DP, RL, DRL	Identify gaps in multi-intersection ATSC	Needs real-world implementation

## Traffic management in V2X Networks

### Traditional traffic management approaches

Conventional traffic management systems, such as static signage and fixed-time signals, remain limited by their inability to account for real-time traffic fluctuations or individual vehicle interactions. These shortcomings often lead to inefficient right-of-way allocation, congestion, and increased accident risks, particularly at intersections^49^. Recent studies highlight the need for more adaptive and intelligent approaches. For example, DRL methods leverage inter-vehicular communication to dynamically coordinate traffic flow, offering a significant improvement over rigid rule-based system^49^. Similarly, research on reversible lanes has underscored the inadequacy of traditional control methods in responding to growing travel demands. The Predictive Empowered Assignment scheme (PEARL) integrates predictive analytics with optimization models to enhance lane assignment, demonstrating the potential of data-driven strategies to outperform static lane control mechanisms in highly developed urban areas^50^.

Other approaches emphasize infrastructure-efficient solutions. Saxena and Adlin proposed a computer-controlled model that uses infrared sensors and detection systems to adjust traffic signals in real time, aiming to reduce congestion and improve safety for both vehicles and vulnerable road users^51^. In a related direction, Tapkir et al. presented an adaptive signal system based on CCTV-enabled traffic density analysis, dynamically adjusting green light durations to balance uneven traffic flows. This method highlights the scalability and cost-effectiveness of reusing existing infrastructure for citywide deployments^52^.

Table 5 provides a comparative overview of these traffic management strategies, illustrating their varying levels of effectiveness in addressing congestion, environmental sustainability, and scalability challenges^53^. Collectively, these studies reveal a broader trend: while emerging technologies such as DRL and predictive analytics promise significant improvements, many proposals remain either domain-specific (e.g., reversible lanes) or limited to pilot-scale implementations (e.g., adaptive signals). A critical challenge remains in integrating these methods into large-scale, heterogeneous traffic networks where robustness, interoperability, and real-time adaptability are essential.

**Table 5:** Comparison of traffic management methods, highlighting their advantages and associated challenges.

**Traffic Control Method**	**Advantages**	**Disadvantages**
Conventional Methods	Decrease traffic congestion and air pollution, cost-effective.	Limited effectiveness, low user satisfaction, increased travel time.
Smart Traffic Signal Systems	Reduce delays and congestion, lower air pollution levels.	High maintenance costs.
Restricted Traffic Zones	Improve air quality, decrease congestion and travel time.	Possible public dissatisfaction may require toll payments.
Advanced Technology Solutions	Enhance traffic efficiency and environmental sustainability.	Higher implementation and maintenance costs.

### Role of V2X in enhancing traffic flow and safety

Expanding on advanced traffic management solutions, recent research emphasizes how CAVs and V2X communication technologies are transforming intersection control, traffic coordination, and road safety. Traditional traffic management methods remain in use, yet their effectiveness can be substantially enhanced through integration with V2I communication^54^. For instance, optimization-based signal controls and traffic-load-responsive reservations supported by CAVs have demonstrated significant efficiency gains—improving throughput by up to 89.63% and reducing waiting times by 60.71% compared with conventional approaches^55^. Similarly, distributed traffic signal control systems leveraging V2X can decrease control delays by 21%, even with only 10% connected vehicle penetration^56^. These findings highlight the scalability of V2X-enabled systems, where even modest adoption rates can yield substantial benefits.

Beyond efficiency, V2X technologies play a critical role in enhancing safety. McNerny et al.^57^ investigated pedestrian safety at crosswalks by embedding V2X antennas in vehicles and mobile devices, showing that strong carrier signal power can be maintained within a 10-meter radius regardless of antenna placement. This ensures reliable vehicle–pedestrian communication, particularly at blind spots and complex intersections, offering a cost-effective pathway to integrating crash-avoidance features into smart city design. Complementing this, Oliva et al.^58^ demonstrated the practicality of IoT-based V2I applications in real-world intelligent intersections in Italy. Their work showed how neural-network-equipped sensors could alert drivers to pedestrians while also enabling rapid passage of emergency vehicles, effectively reducing response times and increasing awareness. Together, these studies underscore how V2X can extend safety benefits beyond vehicles to vulnerable road users (VRUs) and urban emergency systems.

Path planning and traffic flow stability also benefit substantially from V2X integration. Li et al.^59^ introduced a hierarchical co-design framework in which roadside units (RSUs) generate candidate trajectories during pre-planning, while online adjustments account for risk and real-time interactions. This approach improved computational efficiency by 23% and reduced collision rates by 13% compared with conventional methods, reinforcing the role of V2X in enabling scalable real-time planning. In a separate contribution, Li et al.^60^ proposed a cooperative optimization model that integrates vehicle routing and traffic signal scheduling, demonstrating reductions in overall travel time and improved network-level coordination. Similarly, Li et al.^61^ advanced car-following models that incorporate the influence of multiple leading vehicles, showing that field-based influence models can stabilize traffic and mitigate congestion more effectively than distance-based ones. By capturing multi-vehicle dynamics, these models pave the way for active safety technologies capable of reducing collision risks at scale.

Communication reliability remains a major concern for safety-critical V2X applications. Stellwagen et al.^62^ addressed this by combining WLAN-based ITS-G5 with cellular-based LTE-V2X in a hybrid, non-hierarchical framework, achieving improvements in dissemination range, latency, and throughput without requiring additional infrastructure. Nguyen et al.^63^ tackled another limitation—signal shadowing by large vehicles in C-V2X Mode-4. By applying beamforming and relaying strategies, their approach enhanced packet delivery ratios by 117.6% at 500 meters, highlighting the importance of adaptive signal reception and lane-hierarchical strategies in overcoming real-world communication barriers.

Machine learning has further expanded the capabilities of V2X systems. Li et al.^64^ applied an end–edge–cloud architecture to predict vehicle trajectories and selectively disseminate safety messages only to vehicles likely to encounter accident-prone areas. This reduced unnecessary network load while improving relevance. Ribeiro et al.^65^ extended ML applications to VRU safety, training stacked LSTMs to predict collisions involving motorcyclists up to 4.53 seconds in advance with 96% accuracy, though high false positive rates currently limit full automation. These studies illustrate how data-driven intelligence can improve both communication efficiency and predictive safety functions but also emphasize the need for better real-world validation.

Collectively, these contributions show that V2X technologies are not limited to isolated improvements but form a multi-layered ecosystem that transforms the way traffic systems operate. At the infrastructure level, optimization-based intersection management enhances throughput and minimizes delays, demonstrating how even partial deployment of connected vehicles can lead to system-wide benefits. For vulnerable road users, V2X enables applications that extend safety protections beyond drivers, integrating pedestrians, cyclists, and motorcyclists into the traffic management loop. Advanced path planning and traffic coordination frameworks further strengthen stability and efficiency by supporting real-time trajectory adjustments and congestion mitigation. Communication reliability, a persistent challenge in safety-critical environments, is addressed through hybrid and adaptive strategies that ensure robust information exchange under realistic road conditions. Finally, the integration of machine learning introduces predictive capabilities, enabling early detection of risks and proactive safety interventions.

**Fig. 3:**
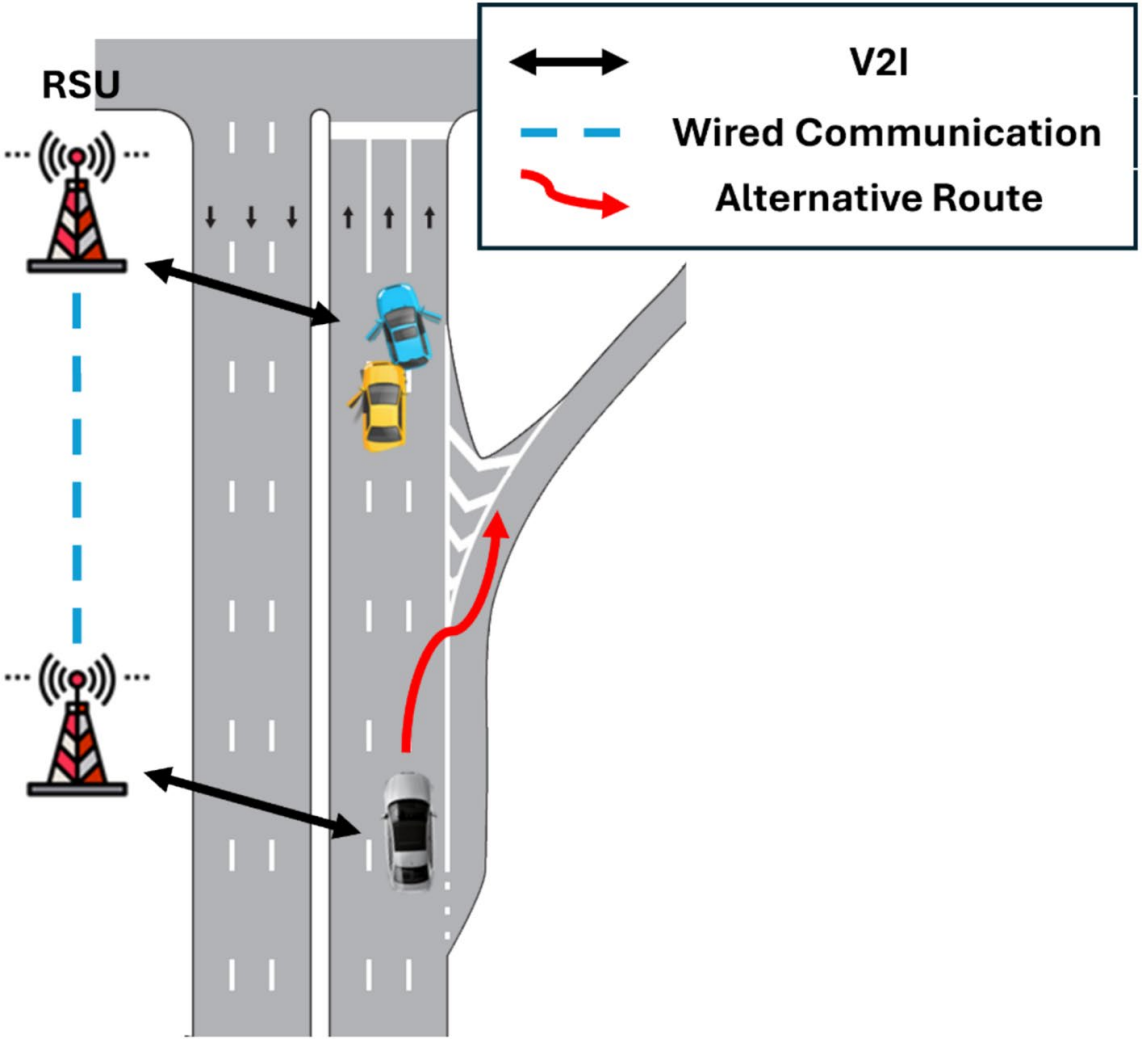
V2X safety message dissemination system.

**Fig. 4:**
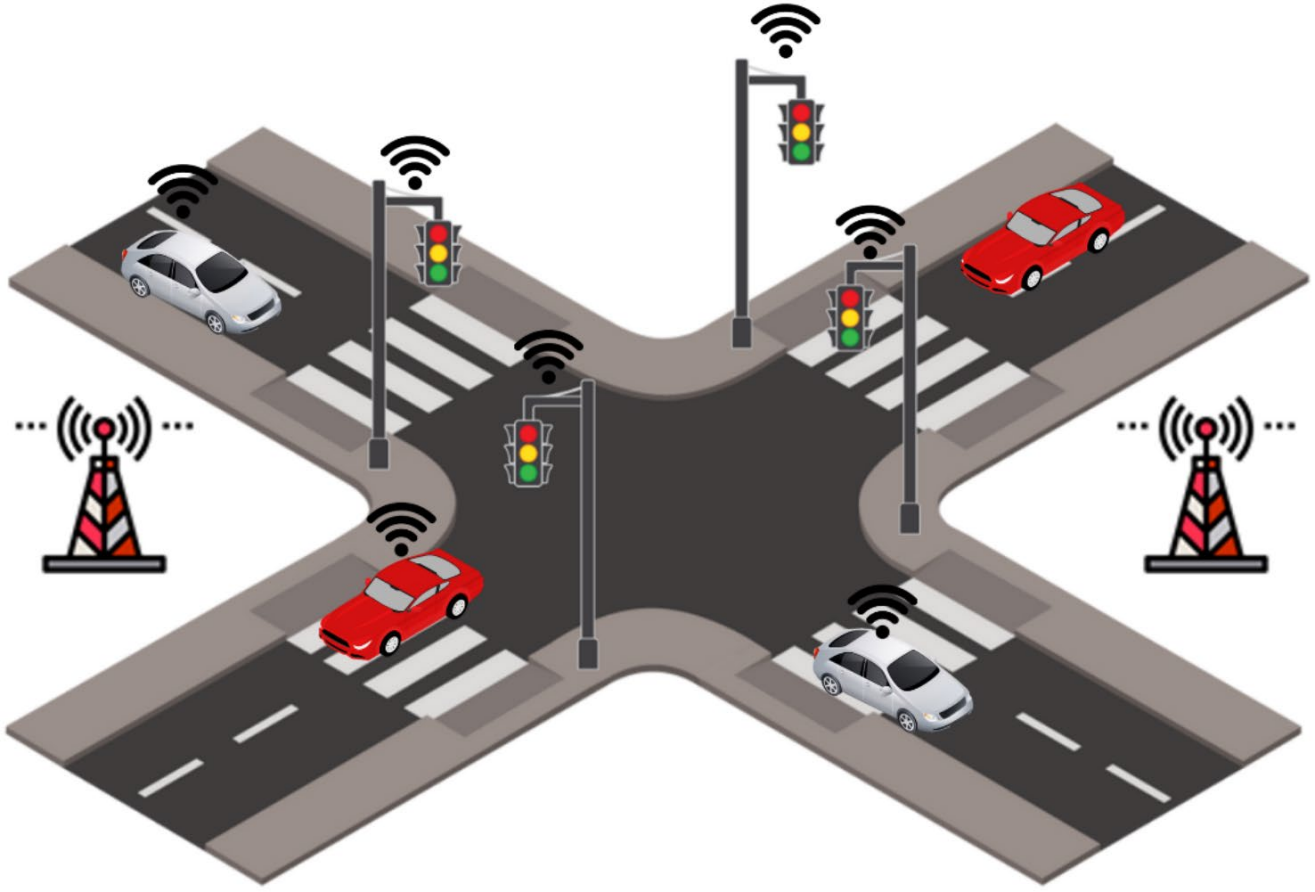
Communication flow between connected vehicles and infrastructure.

Together, these advances highlight how V2X technologies are evolving into the backbone of safer, more efficient, and more resilient Intelligent Transportation Systems, with the potential to reshape urban mobility at scale. A conceptual overview of these interaction flows is illustrated in Fig. 3. and Fig. 4 Together, these advances highlight V2X’s pivotal role in enabling safer, more efficient, and more resilient ITS.

## Accident-aware routing strategies

### Existing accident detection and prediction techniques

Aboulola investigated the use of transfer learning techniques to predict traffic accident severity while addressing the interpretability challenges of deep learning models. The study employs various models, including Multilayer Perceptron (MLP), Convolutional Neural Networks (CNN), Long Short-Term Memory (LSTM), Residual Networks (ResNet), EfficientNetB4, InceptionV3, Extreme Inception (Xception), and MobileNet, with MobileNet achieving the highest accuracy of 98.17%. To enhance transparency and trust in predictive modeling, the study applies Shapley values to analyze the influence of different features on accident severity predictions. By improving both accuracy and interpretability, the research supports evidence-based decision-making for road safety interventions and accident prevention strategies. Future work can extend these findings by refining feature importance analysis and integrating real-time predictive models for proactive traffic management^66^.

Ardakani et al. explored the application of machine learning and big data analysis techniques to predict road traffic accidents and identify key contributing factors. The study proposes a predictive model that preprocesses raw accident data through missing data removal, attribute generalization, and outlier detection using the interquartile method. Four classification models—decision trees, random forest, multinomial logistic regression, and naïve Bayes—are evaluated for accident prediction, with naïve Bayes performing the weakest. The results indicate that accident severity and casualty prediction achieve over 80% accuracy, while vehicle number prediction lags at approximately 64%, possibly due to dataset imbalance. The study highlights the importance of big data frameworks like Apache Spark for handling large-scale accident datasets and suggests integrating advanced techniques, such as neural networks and neutrosophic statistics, to enhance accuracy. Future research directions include incorporating additional environmental factors, balancing dataset proportions, and developing a mobile-based accident prediction and warning system for real-time traffic safety applications^67^.

Alvi et al. presented a critical analysis of existing methodologies for automatic accident detection and prevention, emphasizing the importance of timely emergency response in reducing fatalities. The study reviews various accident detection techniques, including smartphone-based crash prediction, vehicular ad-hoc networks, GPS/GSM-based systems, and machine learning approaches such as neural networks and support vector machines. Additionally, it explores accident prevention strategies, including drowsy and drunk driving detection, speed regulation, and obstacle avoidance using accelerometers, shock sensors, and pressure sensors. While these systems improve road safety by enabling real-time accident detection and emergency service notification, the study highlights a key challenge: the reliance on hardware-based technologies that may fail or provide erroneous readings in severe collisions. Authors suggest the need for more resilient frameworks that minimize dependence on vulnerable sensors and software components. The paper underscores the necessity of integrating robust, fault-tolerant accident detection systems within vehicles to enhance road safety and mitigate traffic hazards effectivel^68^.

**Fig. 5:**
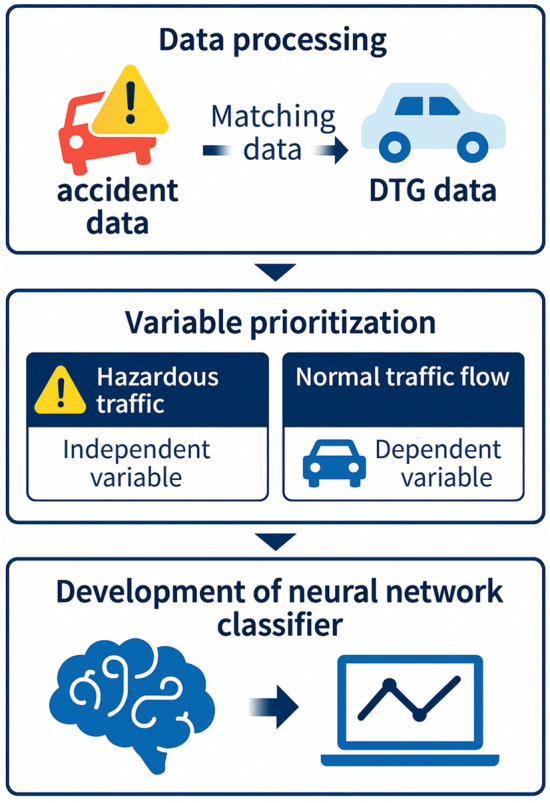
Overall proposed framework.

Kim et al. proposed a proactive accident prevention framework by leveraging digital tachograph (DTG) data to analyze vehicle trajectory patterns on Korean highways. The study moves beyond passive accident response measures, focusing instead on predicting hazardous traffic flows using real-time driving behavior indicators. Through gradient boosting, the top 20 safety indicators influencing traffic flow classification were identified, revealing that dangerous driving events accounted for approximately 33% of studied highway accidents. A neural network-based traffic flow classifier, trained on these indicators, achieved a high accuracy of 94.59%. Furthermore, the study classified DTG data by accident severity, time of occurrence, and weather conditions, with over 90% accuracy in all models. The findings suggest that accident risks increase under adverse conditions, particularly at night and in poor weather. Despite data limitations, the research highlights the potential for real-time crash risk evaluation using tailored safety indicators for different roadway conditions. The study emphasizes the importance of expanding data sources, including passenger vehicle trajectory data, to enhance model reliability and address class imbalance issues in crash prediction. The overall conceptual framework, integrating such proactive prediction mechanisms within a V2X environment, is illustrated in Fig. 5, showcasing the multi-layered architecture for data collection, analysis, and dissemination^69^.

Khosravi et al. applied hierarchical clustering and machine learning techniques to identify accident-prone areas and predict accident severity on the Yazd-Kerman Road in Iran. Using Agglomerative Hierarchical and BIRCH clustering algorithms, the study successfully identified two overlapping accident hotspots, demonstrating high consistency in accident clustering. Field visits, police reports, and interviews with locals revealed key contributing factors: in one area, accidents were linked to a resting area near a mosque, inadequate lighting at curves, and poor road signage; in another, accidents were primarily caused by reduced visibility due to dust storms. Machine learning models—K-Nearest Neighbors (KNN) and Random Forest—were employed to predict accident severity based on environmental and road attributes. KNN outperformed Random Forest with an accuracy of 71% compared to 60%. The study underscores the importance of accurate accident location data and suggests future improvements by incorporating additional variables such as traffic density and road surface conditions. Expanding the dataset to include vehicle types and hospital injury reports is recommended to enhance prediction accuracy and support targeted road safety interventions^70^.

Several machine learning models have been employed for accident prediction, each with varying levels of accuracy and applicability. A comparative analysis of these models is presented in Table 6, detailing their key features, advantages, and limitations.

**Table 6:** Comparison of machine learning models for accident prediction.

**Study**	**Model**	**Accuracy (%)**		**Key Features**	**Strengths**	**Limitations**
Aboulola^66^	MobileNet		98.17	Deep learning, CNN-based	High accuracy	Computationally expensive
	Decision Tree		85.4	Feature-based classification	Interpretability	Prone to overfitting
Ardakani et al.^67^	Random Forest		87.6	Ensemble learning approach	Robust performance	Slower inference time
	Naïve Bayes		60.0	Probabilistic classification	Fast computation	Low prediction power
Kim et al.^69^	Neural Network		94.59	Gradient boosting, DTG data	High predictive power	Require large dataset
Khosravi et al.^70^	K-Nearest Neighbors (KNN)		71.0	Distance-based classification	Simple to implement	Lower accuracy
	Random Forest		60.0	Road & environmental attributes	Identifies accident hotspots	Limited dataset size

### Impact of accidents on route optimization

The optimization of routing strategies for accident mitigation has been addressed from multiple perspectives. For hazardous materials (hazmat) transport, Song et al.^71^ proposed a bi-objective rail–truck routing model to minimize risks and costs under real-world constraints such as traffic restrictions and train schedules. Using the Max-Min Ant Colony Algorithm (MMAS), their case study in the Beijing–Tianjin–Hebei region showed that traffic restrictions increase risks by 4.2%–9.13% and costs by 3.32%–5.25%, while alternative national highway routes reduce risks by 9.1%. Extending this direction, Liu et al.^72^ incorporated equity considerations among stakeholders by integrating emergency response times and compensation mechanisms into the risk assessment. Their genetic algorithm, tested on Shanghai data, demonstrated that accounting for fairness produces safer and more balanced hazmat routes.

While hazmat routing emphasizes long-term planning, emergency response routing requires real-time adaptability. Luan et Jiang^73^ developed a mixed-integer linear programming model with semi-soft time windows (MIPSSTW) to optimize ambulance dispatch under time-varying traffic conditions. Their modified cuckoo search (MCS) algorithm improved convergence and reduced EMS delays, as validated with real-world Chinese data. Complementarily, Wen et al.^74^ introduced a Timing Co-Evolutionary Path Optimization (TCEPO) method that dynamically adapts rescue routes based on predicted traffic states. Simulation results revealed travel time reductions of 17.65%–40.02% compared to CEPO and 26.34%–38.47% compared to OLRO, highlighting its potential for rapid and reliable emergency response.

Beyond route-level decisions, infrastructure-based strategies have also been explored. Zhang et al.^75^ addressed accident-prone intersections by proposing an accident-risk-based Roadside Unit (RSU) deployment framework. Using AHP and entropy weighting to assess risks across road, accident, and environmental dimensions, they applied an improved 0–1 knapsack algorithm to optimize RSU placement. SUMO and Veins simulations confirmed that their approach achieved 2.63%–2.86% higher vehicle coverage, 5.04% better accident coverage, and 5.72% higher accident-risk coverage than traditional RSU deployment methods.

Finally, structural characteristics of road networks themselves have been linked to accident risk. Li et al.^76^ applied a Segment Analysis (SA)-Apriori model to geospatial data from Chongqing’s Dadukou District, revealing that roads with high global integration and medium-to-high global choice are strongly correlated with major RTAs, while minor accidents showed no such correlation. This suggests that network topology should be explicitly integrated into route optimization to mitigate severe accident risks.

In summary, these studies illustrate a continuum of accident mitigation strategies: hazmat routing models (Song et al.^71^, Liu et al.^72^) address risk and fairness in dangerous goods transport, emergency vehicle routing methods (Luan et Jiang^73^, Wen et al.^74^) ensure fast and adaptive responses, infrastructure-based approaches (Zhang et al.^75^) enhance safety at intersections, and spatial analyses (Li et al.^76^) provide insights into how road network design affects accident likelihood. Together, they highlight the multi-layered nature of accident-aware route optimization.

To provide a clearer comparison, Table 7 summarizes the reviewed route optimization methods for accident mitigation, outlining their methodologies, features, performance metrics, advantages, and limitations.

**Table 7:** Comparison of route optimization methods for accident mitigation.

**Study**	**Optimization Method**	**Key Features**	**Performance Metrics**	**Advantages**	**Limitations**
Song et al.^71^	Bi-objective Mathematical Model (MMAS Algorithm)	- Minimizes transportation risk & cost - Considers traffic restrictions & alternative routes - Time-varying risk factors included	- Risk increase: 4.2%–9.13% - Cost increase: 3.32%–5.25% - Risk reduction by 9.1% using alternative routes	- Accounts for real-world constraints - Improves coordination between rail & road transport	- Does not incorporate dynamic real-time data - Focuses mainly on hazardous freight transportation
Luan et Jiang^73^	Mixed-Integer Linear Programming (MIPSSTW) & Modified Cuckoo Search (MCS)	- Optimizes emergency vehicle routing - Accounts for traffic flow & intersections - Uses improved Bureau of Public Roads (BPR) model	- Reduces EMS response time - Solves high-dimensional optimization problems	- Adaptable to dynamic traffic conditions - Improved accuracy with Lévy flight strategy	- High computational complexity - Limited applicability for large-scale multi-depot problems
Zhang et al^75^.	0-1 Knapsack Algorithm for RSU Deployment	- Uses accident risk as key factor - Integrates AHP & Entropy Weight Method (EWM) for risk assessment	- 2.63%–2.86% improvement in vehicle coverage - 5.04% increase in accident coverage - 5.72% improvement in risk coverage	- Effectively enhances intersection safety - Accounts for environmental & traffic conditions	- Deployment may be cost-intensive - Requires accurate risk data for optimal placement
Liu et al.^72^	Multi-Objective Genetic Algorithm (Risk-Equity-Based Optimization)	- Balances transportation risk, cost & risk equity - Considers emergency response time	- Equitable distribution of risk - Enhanced safety in hazmat transportation	- Ensures fair risk allocation - Incorporates government & carrier concerns	- Requires compensation mechanisms for implementation - Needs real-time traffic integration
Wen et al.^74^	Timing Co-Evolutionary Path Optimization (TCEPO)	- Dynamic path optimization - Uses Ripple Spreading Algorithm (RSA) for real-time updates	- Reduces travel time by 17.65%–40.02% vs. CEPO - 26.34%–38.47% reduction vs. OLRO	- Continuously adapts to traffic changes - Highly effective for emergency rescues	- Computationally intensive - Needs continuous real-time traffic updates
Li et al.^76^	Segment Analysis (SA)-Apriori Model	- Examines spatial characteristics of road networks - Correlates road features with accident rates	- High global integration roads linked to RTAs - Major accidents occur on high-choice roads	- Helps in proactive route planning - Can improve urban safety policies	- Limited to urban network analysis - Does not incorporate real-time traffic data

## Algorithms for route optimization

Within V2X systems, the A* algorithm enables vehicles to make informed decisions by processing real-time traffic data and identifying potential hazards. When combined with artificial intelligence (AI), A* supports predictive accident management and optimized route planning, enhancing both driver awareness and safety^77^. In autonomous vehicles, this integration has proven effective for real-time navigation, obstacle avoidance, and optimal route selection, thereby contributing to safer and more efficient road networks^78^.

Although A* was originally developed for static environments, significant improvements have extended its applicability to dynamic traffic conditions. One such enhancement is the Asymptotically Optimal A* (AOA*), designed for kinodynamic planning in continuous spaces. By integrating heuristic functions and advanced pruning techniques, AOA* improves motion planning under dynamic conditions^79^. Similarly, Liu et al. proposed the Multi-Search Strategy A* (MSSA*) to address inefficiencies in high-complexity 3D environments such as offshore pipe routing. Key innovations include a node directional discrimination rule to reduce redundancy, a double-layer domain extension for broader exploration, a multi-factor heuristic evaluation function, and dynamic adaptive weighting. These improvements achieved higher efficiency and accuracy than conventional A* while maintaining flexibility across diverse routing scenarios^80^.

**Fig. 6:**
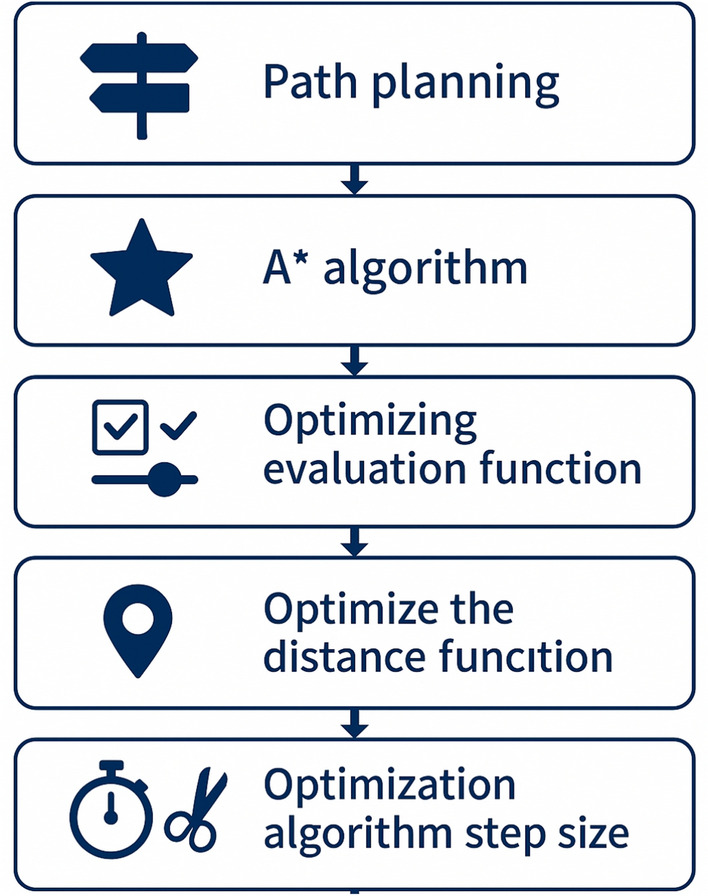
Block diagram of algorithm optimization.

Further refinements have been made by Sang et al.**,** who introduced Directional Search A* to improve path planning by addressing sharp turns, weak directional guidance, and excessive node computations. By incorporating an angle constraint into the evaluation function, optimizing distance guidance, and adjusting step sizes, this method generated smoother and shorter paths while reducing planning time. As illustrated in Fig. 6, simulation results demonstrated improved smoothness and efficiency compared to traditional A*. Future work aims to further enhance adaptability by dynamically adjusting step sizes based on obstacle size in unknown environments^81^.

Sui et al. proposed a congestion-aware A* enhanced with a spatio-temporal graph convolutional network (ST-GCN), which models traffic congestion events and dynamically predicts travel times. The integration of A* with a path-aided neural network demonstrated superior performance on real-world datasets, improving accuracy and efficiency in dynamic routing^82^. Zhang et al. introduced a hybrid algorithm combining A* with bidirectional RRT, where A* provides a coarse global path and RRT refines it under vehicle chassis constraints. This hybrid approach significantly reduced path length and computation time, achieving up to an 1800% speed increase in complex environments^83^. Yan et al. improved A* for ITS applications by integrating minimum heap sorting and bidirectional hierarchical search strategies, which enhanced search speed and practical route design. Comparative results confirmed its superiority over classical algorithms such as Dijkstra and Bellman-Ford, particularly in large-scale urban navigation^84^. Sang et al. developed Directional Search A*, incorporating an angle constraint and adaptive step size optimization to produce smoother trajectories with reduced computation. While effective, future work must focus on adapting step sizes dynamically to varying obstacle environments^81^.

Beyond A* variants, Ru explored multimodal logistics optimization using a graph traversal algorithm combined with Tabu search. The approach minimized transportation costs and improved profitability, outperforming genetic algorithms and simulated annealing in terms of accuracy, though at the expense of longer computation time. These findings highlight the potential of metaheuristic algorithms such as Tabu search for complex vehicle routing and intermodal transport optimization^85^.

**Fig. 7:**
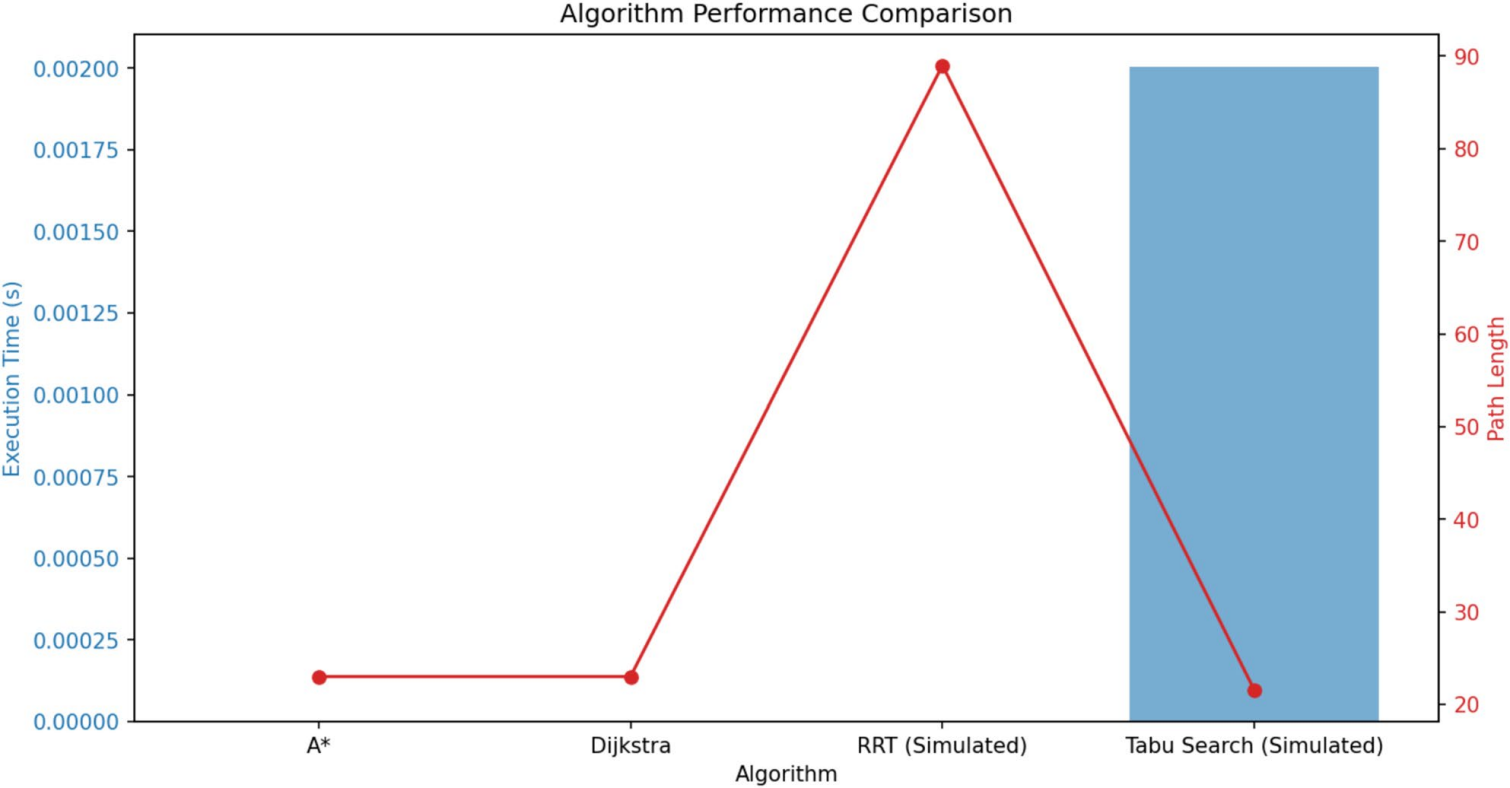
Algorithm performance comparison in terms of execution time and path length for different route optimization approaches in V2X-based traffic management.

To evaluate the efficiency of various search algorithms in accident-aware route optimization, we compare their execution time and path length. As shown in Fig. 7 Algorithm performance comparison in terms of execution time and path length for different route optimization approaches in V2X-based traffic management., A* and Dijkstra’s algorithms produce the shortest paths, whereas RRT and Tabu Search exhibit higher path length variations. However, A* outperforms other methods in terms of execution time.

Recent works have demonstrated the strength of metaheuristic approaches in solving complex vehicle routing problems (VRPs). Liu et al. developed the SFSSA algorithm, combining chaotic mapping, sine cosine optimization, and firefly-based perturbation to improve solution diversity and convergence, achieving superior performance across Solomon benchmark cases^86^. Similarly, Korzeń et al. applied Ant Colony Optimization (ACO) to tram route planning, demonstrating practical gains in public transport efficiency but highlighting limitations in handling infrastructure and accessibility constraints^87^. For electric vehicle logistics, Bezzi et al. introduced a branch-and-price formulation for the EVRP with partial recharges, showing scalability to multiple charging technologies^88^, while Wang et al. proposed a two-phase evolutionary algorithm for electric location-routing optimization, improving charging station placement^89^. Together, these studies illustrate the adaptability of metaheuristics for multimodal, electric, and public transport routing, though challenges remain in balancing solution quality with computational efficiency under real-time conditions.

Graph traversal methods remain central to accident-aware traffic optimization. Lu et al. proposed One-Way Search (OWS) for multi-request route planning in point-of-interest networks, outperforming greedy methods through advanced pruning and iterative refinement^90^. Ma et al. combined DFS with genetic algorithms for railway scheduling, reducing conflicts between train operations^91^, while Qi et al. and Liu et al. leveraged BFS variants for connectivity testing in ring networks and formation planning in multi-robot systems, respectively^92,93^. These approaches demonstrate the efficiency of graph-based search in constrained environments, though scalability to dynamic and stochastic traffic remains an open challenge.

Several studies propose hybrid or domain-specific approaches. Zhan et al. modeled route planning under severe weather as a Markov decision process, integrating BFS with Edmonds’ algorithm and Fibonacci heaps for improved reachability analysis^94^. Zheng et al. optimized BFS detection for large-scale MIMO systems, achieving significant complexity reduction^95^. Hongjie et al. integrated clustering, trajectory smoothing, and Dijkstra’s algorithm for ship routing, balancing safety and efficiency^96^. Zhang et al. explored corrugated box transport optimization under demand uncertainty using arc-flow formulations and branch-and-price^97^. While domain-specific, these studies highlight the growing need for hybrid frameworks that incorporate environmental, infrastructural, and stochastic factors.

A growing trend in vehicle routing research is the integration of machine learning and reinforcement learning with classical optimization. Wang et al. combined self-supervised reinforcement learning with the LKH heuristic, improving generalization and solution quality across VRP variants^98^. Similarly, Hussain et al. introduced OptiE2ERL, an RL-based approach for energy-efficient routing in IoV, which dynamically balances residual energy, bandwidth, and mobility, outperforming classical algorithms such as LEACH and PEGASIS^99^. Wang et al. further applied DRL-enhanced Adaptive Large Neighborhood Search (ALNS) to the Capacitated EVRP, improving charging-aware route optimization^100^. Beyond RL, Jiang et al. demonstrated the potential of graph neural networks (GNNs) for routing, though challenges of scalability, interpretability, and security remain^101^. Together, these studies highlight the shift toward data-driven routing, though widespread deployment requires advances in explainability, online adaptability, and access to large-scale real-world datasets.

Metaheuristic innovations continue to enrich vehicle and VANET routing. Soundarayaa and Balasubramanian developed the Komodo Mlipir Algorithm-based KMAORDM to reduce latency and overhead in VANETs, achieving measurable improvements in throughput^102^. Alqahtani and Kumar applied hybrid metaheuristics to EnFV routing, demonstrating potential for multi-objective optimization in emerging mobility systems^103^. These approaches emphasize exploration–exploitation balance and multi-objective adaptability but share challenges of computational efficiency and parameter sensitivity.

Optimization-based frameworks provide additional perspectives. Scroccaro et al. introduced inverse optimization (IO) to learn human routing preferences, achieving competitive performance in the Amazon Last Mile Routing Challenge^104^. Yang et al. used MILP to compare time-based vs. quantity-based delivery consolidation, offering insights into when stability or flexibility is preferable in supply chains^105^. Wang et al. extended this to collaborative multi-depot VRPs with dynamic customer demands, underscoring the complexity of balancing cost, adaptability, and computational feasibility^106^. Song and Cheng advanced a mean–standard deviation routing model for congestion-prone environments but highlighted computational scalability as an enduring limitation^107^. These works illustrate how mathematical programming, and IO can capture realistic decision-making trade-offs but require hybridization with learning-based methods for real-time adaptability.

Graph-based methods continue to underpin routing research, particularly in specialized domains. Li et al. highlighted the challenges of alternative route generation across platforms such as Google Maps, where subjectivity and inconsistent data sources complicate evaluation^108^. Scheffler explored the structural complexity of BFS/DFS search trees, revealing computational challenges in determining feasible leaf nodes^109^. Although more theoretical, these contributions underline persistent gaps in bridging graph-theoretic insights with practical V2X routing systems.

A summary of recent routing algorithms and their performance metrics is presented in Table 8 Comparative analysis of routing algorithms.. The table compares methodologies such as A* variants (e.g., MSSA*, Directional Search A*), bio-inspired algorithms (e.g., ACO, SFSSA), and hybrid approaches (e.g., A* + RRT, Hybrid DFS + GA). Key observations include:

- **Fast execution times** are achieved by heuristic-driven methods (e.g., Enhanced A*, Optimized BFSD), though some require higher computational resources (e.g., Congestion-aware A*).
- **Path length optimization** is a common strength, particularly in algorithms incorporating dynamic weighting (e.g., MSSA* or real-time traffic prediction (e.g., ST-GCN).
- **Limitations** include adaptability challenges (e.g., Tabu Search’s slow runtime) and dependency on structured environments (e.g., DFS ’s network constraints).
- This synthesis aids in selecting context-appropriate algorithms for applications like autonomous vehicles, logistics, and IoT networks.

**Table 8:** Comparative analysis of routing algorithms.

**Study**	**Algorithm**	**Key Features**	**Execution Time**	**Path Length**	**Advantages**	**Limitations**
^79^	AOA*	Kinodynamic planning, heuristic functions	Fast	Short	Suitable for dynamic traffic conditions	Complex implementation
^80^	MSSA*	Multi-search strategy, dynamic weighting	Moderate	Short	Efficient in 3D routing scenarios	Needs further adaptability research
^81^	Directional Search A*	Angle constraints, optimized distance function	Fast	Short	Reduces sharp turns and redundant nodes	Needs dynamic step-size adjustment
^82^	Congestion-aware A*	ST-GCN for real-time traffic prediction	Fast	Short	Accurate for dynamic traffic conditions	High computational demand
^83^	A* + RRT	Two-level mapping, Bezier curve smoothing	Very Fast	Shorter	Significant speed improvement over RRT	Map transitions need optimization
^84^	Enhanced A*	Min-heap sorting, bidirectional search	Very Fast	Short	Efficient for ITS applications	Needs real-time adaptability refinement
^85^	Tabu Search	Multimodal transport route optimization	Slow	Variable	Low cost per km, high profit increase	High running time
^86^	SFSSA	Sine cosine + firefly perturbation	Fast	Short	Optimal for VRPSPDTW problems	Needs carbon emissions considerations
^87^	ACO	Optimization of tram routes	Moderate	Short	Effective for public transport planning	Insensitive to local infrastructure
^89^	Multi-objective Evolutionary Algorithm	Interpolation + surrogate modeling	Fast	Short	Optimized charging station placement	Needs real-time data integration
^90^	OWS	Branch-and-bound + greedy search	Fast	Short	Outperforms state-of-the-art MRRP algorithms	NP-hard problem complexity
^91^	Hybrid DFS + GA	Railway station optimization	Fast	Short	Improves punctuality and efficiency	Needs real-time dynamic adjustments
^92^	DFS	Efficient edge testing in ring networks	Fast	Short	Optimized for connectivity detection	Limited to structured networks
^94^	BFS	Markov decision-based route planning	Moderate	Variable	Handles stochastic conditions well	High computational overhead
^93^	BFS for MMRS	Motion planning with formation constraints	Fast	Short	Effective in obstacle-rich environments	Struggles in highly dynamic scenarios
^95^	Optimized BFSD	Monte Carlo-based width optimization	Very Fast	Short	Reduced complexity in large MIMO systems	Managing layer width remains a challenge
^96^	Dijkstra	Polynomial Approximation for ship routing	Fast	Short	Improves trajectory similarity and efficiency	Computational cost of large datasets
^88^	Branch-and-Price	EVRP with multi-recharge options	Fast	Short	Efficient for depot-to-depot optimization	Complex stabilization techniques needed
^104^	Inverse Optimization	Learning decision-maker preferences	Fast	Short	Real-world applicability	Requires extensive historical data
^98^	SSRL	RL + LKH heuristic	Fast	Short	Superior accuracy and efficiency in VRP	Needs broader combinatorial optimization testing
^102^	KMAORDM	Komodo Mlipir Algorithm for VANETs	Fast	Short	Reduces latency, improves QoS	Needs adaptability for urban scenarios
^99^	OptiE2ERL	RL-based energy-efficient routing	Fast	Short	Extends network lifetime, reduces overhead	Needs further validation in large-scale IoV
^105^	Time-Based VRP	Consolidation strategies for delivery	Moderate	Short	Cost-efficient in stable markets	Limited adaptability for volatile demand

## Discussion

Over the past decade, several survey articles have been published on V2X communication and intelligent transportation systems. However, most of these studies adopt a broad scope, focusing on general V2X technologies, ITS architectures, or AI-based traffic management, without emphasizing the integration of accident detection, accident-aware routing, and real-time optimization within V2X-enabled networks. For instance, Yogarayan et al.^110^ compared DSRC and C-V2X technologies but did not examine accident-aware routing, while Hamdi et al.^112^ reviewed accident detection techniques without linking them to optimization strategies. Zulkarnain and Putri^111^ used NLP methods to map ITS research but did not highlight accident management, and Elassy et al.^114^ emphasized sustainability without proposing a research roadmap. Table 9 highlights these limitations, contrasting them with the more focused contribution of this review. While prior surveys address V2X and ITS at a high level, they are fragmented, confirming the need for a comprehensive synthesis explicitly linking accident detection, routing, and optimization within V2X networks.

**Table 9:** Comparison of related survey articles and their limitations.

**Reference**	**Year**	**Contribution**	**Limitations**
Yogarayan et al.^110^	2021	Comparative review of DSRC, C-V2X, and hybrid approaches; discussion of platforms, products, and deployment challenges	No coverage of accident management or routing optimization
Zulkarnain et Putri^111^	2021	Systematic review using NLP methods to map ITS research, trends, and knowledge growth	Broad ITS review, but no specific focus on accident management or V2X-based routing optimization
Hamdi et al.^112^	2021	Review of incident detection technologies and algorithms within VANET environments	Narrow scope; lacks connection to routing and optimization
Zemmouchi-Ghomari^113^	2025	Comprehensive review of AI applications in ITS, covering architectures, benefits, challenges, and case studies	No discussion of V2X communication standards
Elassy et al.^114^	2024	Broad review of ITS components (VANETs, intelligent/virtual traffic lights, mobility prediction) and communication systems with sustainability focus	Limited discussion of accident-aware routing; lacks research roadmap
This Article	2025	Comprehensive synthesis of communication, ITS, detection, routing, and optimization for accident-aware systems; addresses gaps by linking fragmented domains into a unified framework, while highlighting open challenges and proposing a forward-looking research roadmap	

The analysis of existing literature on accident-aware traffic management within V2X networks reveals both significant progress and persistent shortcomings. While numerous studies have explored communication protocols, accident detection methods, and optimization algorithms, the findings indicate that these efforts are often fragmented and evaluated in isolation. This section critically synthesizes the reviewed works, emphasizing overarching trends, persistent challenges, and directions for future research. Although strong advances exist in each subdomain, their integration into a unified accident-aware V2X ecosystem is still underdeveloped.

### V2X communication technologies: strengths and gaps

Current V2X communication technologies, namely DSRC and C-V2X, have demonstrated significant potential in enabling low-latency, reliable message exchange, which is indispensable in accident-aware traffic management. DSRC provides latency as low as 10 ms in field tests^27,28^, making it effective for safety-critical messaging, while C-V2X over 5G supports throughputs up to 10 Gbps and long-range mobility^31,32^. These capabilities are summarized in Table 2 and Table 3, while their operational modes are depicted in Figure 1. However, these technologies continue to evolve in parallel rather than converging, creating fragmentation and compatibility challenges across heterogeneous vehicular environments^31^.

A recurring limitation in the existing literature is the predominance of simulation-based performance analyses, which typically focus on latency, throughput, and packet delivery ratio. While valuable, they lack large-scale real-world validation. For example, most DSRC studies rely on small-scale testbeds, and C-V2X evaluations remain largely simulation-based, leaving open questions about performance under urban density, high mobility, multipath interference, and unpredictable communication failures.

Security and reliability are persistent concerns. V2X systems remain vulnerable to spoofing, denial-of-service, and data manipulation attacks. While cryptographic methods and intrusion detection frameworks have been proposed, their scalability under real-time vehicular conditions remains uncertain^17,33^. Moreover, imperfections such as packet loss and message delay significantly degrade the performance of autonomous intersection control and cooperative driving^34^.

Future research must therefore prioritize hybrid frameworks that integrate DSRC, C-V2X, and emerging 5G/6G technologies into interoperable systems. Embedding adaptive AI-driven communication management can help balance latency, bandwidth, and security in real time, while sustainability considerations—such as energy efficiency in 6G-enabled vehicular networks—will ensure long-term feasibility. DSRC and C-V2X have laid the groundwork for accident-aware V2X, but widespread adoption depends on solving interoperability, cybersecurity, large-scale validation, and sustainability challenges.

### ITS and V2X integration

The integration of ITS with V2X communication represents a paradigm shift from reactive traffic management to adaptive, predictive, and safety-oriented mobility. Studies confirm that even modest penetration rates of connected vehicles improve efficiency and safety. For example, distributed V2X-enabled traffic signals reduce control delays by 21% at just 10% penetratio^56^. Reinforcement learning–based Adaptive Traffic Signal Control (ATSC) reduces delays by 38% and fuel consumption by 4.5%^40^, while graph neural networks improve coordination across multiple intersections^42^. These innovations are compared in Table 4, with architectures shown in Fig. 2.

Hybrid and adaptive communication strategies also enhance performance. Integrating ITS-G5 with LTE-V2X improves dissemination range by 21% and mitigates shadowing by large vehicles^62^. Cooperative frameworks that link vehicle routing with adaptive signal control further reduce congestion and travel time.

Machine learning is central to ITS–V2X integration. Reinforcement learning supports adaptive multi-agent control, while end–edge–cloud frameworks reduce communication loads by selectively broadcasting safety-critical data. Predictive models have also achieved high accuracy in collision forecasting, offering proactive protection for VRUs and motorcyclists^65^.

However, challenges remain computational cost, interoperability, and security vulnerabilities hinder deployment. Large-scale pilot testing under real urban conditions is still rare. ITS–V2X integration has demonstrated clear benefits in delay reduction, fuel efficiency, and accident prevention, but real-world scaling requires hybrid communication, interoperability, and city-scale pilots.

### Accident detection and prediction: toward explainable AI

Machine learning and deep learning models show strong performance in accident detection and prediction. CNNs and LSTMs achieve accuracies exceeding 95% on benchmark datasets^65^, while MobileNet achieves 98.17% accuracy in accident severity classification^66^. Gradient boosting on digital tachograph data achieves 94.59% accuracy in crash risk prediction^69^. Clustering methods, such as BIRCH, identify accident hotspots based on historical crash records^70^. Comparative results are summarized in Table 6, with conceptual frameworks shown in Fig. 5.

Despite these successes, two major limitations constrain adoption. First, most models rely on limited, often imbalanced datasets, raising questions about generalizability across regions. Second, interpretability is lacking DL models operate as “black boxes,” limiting user trust in safety-critical settings.

Recent studies have explored Explainable AI (XAI) techniques, such as Shapley values, which clarify which factors most influence predictions^66^. Federated learning has also been proposed to enable collaborative model training across multiple regions without violating privacy.

Looking ahead, integrating heterogeneous data sources—including trajectories, weather, and infrastructure data—into multimodal frameworks could reduce bias and improve robustness. Lightweight, edge-compatible models will also be essential for real-time deployment in roadside or vehicular units. ML/DL-based accident prediction holds great promise, but progress depends on interpretability, multimodal data fusion, and deployment-ready lightweight models.

### Accident-aware routing and optimization strategies

Accident-aware routing and optimization are essential for resilience and safety in V2X systems. Traditional algorithms like Dijkstra and A* remain foundational, with enhanced A* variants reducing computation times by up to 1800% compared with RRT in complex environments^83^. These results are presented in Table 8.

Metaheuristic methods, including Ant Colony Optimization (ACO), Tabu Search, and hybrid evolutionary approaches, extend adaptability to multimodal routing. For example, ACO has been applied to optimize tram scheduling^87^, while genetic algorithms improve fairness in hazardous material routing^72^.

Hybrid frameworks—such as combining A* with RRT—yield smoother trajectories, and congestion-aware methods enhanced with spatio-temporal graph convolutional networks (ST-GCN) anticipate future traffic states for proactive routing^82^. Reinforcement learning–based frameworks, such as OptiE2ERL, optimize multi-objective performance, balancing travel time, energy use, and collision risk.

Despite advances, many models assume perfect communication and overlook latency, packet loss, and cybersecurity risks. Most also prioritize travel time while neglecting sustainability or equity. Accident-aware routing is progressing toward multi-objective, AI-enhanced frameworks, but practical deployment requires addressing communication imperfections, computational scalability, and validation in realistic traffic environments.

### Cross-cutting challenges and critical reflections

While accident-aware V2X systems have achieved notable progress across communication, detection, and routing, several overarching challenges continue to constrain their large-scale deployment and real-world applicability. These challenges are technical, societal, and regulatory in nature, underscoring the need for holistic solutions that go beyond algorithmic advances.

Lack of standardized datasets and benchmarks. A persistent limitation in the field is the scarcity of open-access, accident-aware traffic datasets. Most existing studies depend on small-scale simulations or proprietary data, which restricts reproducibility and makes fair benchmarking difficult^110,112^. Without standardized datasets, it is challenging to evaluate generalizability across diverse traffic and environmental conditions. Initiatives such as the HighD and NGSim datasets have advanced trajectory analysis but remain limited in accident-related labeling. Establishing large-scale, open, and representative accident-aware datasets is a prerequisite for robust evaluation, as highlighted in Table 9.

Cybersecurity and privacy risks. Accident-aware V2X systems rely on continuous data exchange between vehicles, infrastructure, and cloud platforms, exposing them to threats such as spoofing, denial-of-service, and data manipulation. Studies have demonstrated that message injection attacks can increase collision probability by over 30% in simulated networks^17^. Blockchain and distributed ledger technologies have been proposed to secure V2X data integrity^33^, yet these methods introduce added latency (up to 20–30% overhead) and computation costs that limit scalability in real-time scenarios. Furthermore, privacy concerns remain acute given the sensitivity of location and driver behavior data.

Scalability and computational efficiency. Advanced optimization and learning frameworks often demonstrate strong results in controlled conditions but degrade in dense urban environments. For example, deep reinforcement learning approaches require billions of training iterations to converge and can experience a 40–60% performance drop when scaled to high-density traffic^82^. Hybrid heuristics also suffer from exponential growth in computation time as network size increases. Edge computing and lightweight model compression have been suggested as solutions, but empirical validation in real-world vehicular testbeds is limited.

Ethical and societal considerations. Beyond technical concerns, accident-aware systems raise unresolved ethical dilemmas. Autonomous decision-making during unavoidable collisions often requires prioritizing between occupants, pedestrians, or vulnerable road users (VRUs). While some studies propose utilitarian frameworks for “least harm” decision-making, consensus on how to operationalize such values in practice remains absent. Moreover, equity concerns persist, as deployment tends to favor technologically advanced regions, potentially widening safety gaps between urban and rural areas. Addressing these issues will require not only engineering innovation but also regulatory alignment and multidisciplinary collaboration among engineers, ethicists, and policymakers.

The advancement of accident-aware V2X systems is contingent upon overcoming systemic challenges in dataset availability, cybersecurity, scalability, and ethical governance. Progress in these domains is as critical as algorithmic innovation, ensuring that V2X evolves into a safe, trustworthy, and socially equitable mobility ecosystem.

## Conclusion and future directions

This review has examined advancements in V2X-enabled accident-aware traffic management, with a particular emphasis on routing and optimization strategies. The surveyed literature demonstrates how search algorithms, metaheuristics, and AI-driven approaches contribute to real-time navigation, congestion mitigation, and safety improvements. V2X communication has emerged as a transformative enabler, supporting cooperative decision-making among vehicles, infrastructure, and vulnerable road users. Despite these advances, significant challenges remain, including latency in communication, cybersecurity vulnerabilities, scalability in dense urban networks, and unresolved ethical questions in accident decision-making. Collectively, these findings underscore both the promise and the limitations of current research, highlighting the need for more integrated and robust frameworks.

Looking forward, future research should address these challenges by combining classical optimization with advanced machine learning to improve adaptability and predictive accuracy in dynamic traffic environments. Edge and fog computing architectures will be essential to minimize latency and computational bottlenecks, while blockchain-based security and AI-driven intrusion detection can strengthen data integrity and resilience against cyber threats. Standardized open datasets and real-world testing are also critical to evaluate scalability and ensure the practical deployment of proposed solutions. Finally, future work must engage more deeply with ethical and societal considerations, particularly in mixed traffic scenarios involving both human-driven and autonomous vehicles. By tackling these areas, accident-aware V2X systems can move closer to realizing their potential as the backbone of safer, more efficient, and more sustainable transportation networks.

## Data Availability

This review article does not involve the generation or analysis of new data. However, any materials, references, or resources used in the review are cited within the manuscript. For further information or requests regarding the data or materials referenced in this study, please contact the corresponding author at: hossamzohir@std.mans.edu.eg.

## Abbreviations

V2X: Vehicle-to-Everything
V2P: Vehicle-to-Pedestrian
DFS: Depth-First Search
BFS: Breadth-First Search
ITS: Intelligent Transportation Systems
VEINS: Vehicles in Network Simulation
VANETs: Vehicular Ad-Hoc Networks
V2V: Vehicle-to-Vehicle
V2I: Vehicle-to-Infrastructure
SUMO: Simulation of Urban MObility
DRL: Deep Reinforcement Learning
RL: Reinforcement Learning
DSRC: Dedicated Short-Range Communication
C-V2X: Cellular-V2X
